# Micro-ultrasound Versus Magnetic Resonance Imaging in Prostate Cancer Active Surveillance

**DOI:** 10.1016/j.euros.2022.09.019

**Published:** 2022-10-25

**Authors:** Patrick Albers, Betty Wang, Stacey Broomfield, Anaïs Medina Martín, Christopher Fung, Adam Kinnaird

**Affiliations:** a Division of Urology, Department of Surgery, University of Alberta, Edmonton, Canada; b Alberta Prostate Cancer Research Initiative, Edmonton, Alberta, Canada; c Department of Radiology & Diagnostic Imaging, University of Alberta, Edmonton, Canada; d Cancer Research Institute of Northern Alberta, Edmonton, Canada; e Alberta Centre for Urologic Research and Excellence, Edmonton, Canada; f Department of Oncology, University of Alberta, Edmonton, Canada

**Keywords:** Prostate Cancer, Prostate biopsy, Micro-ultrasound, Active surveillance, Targeted biopsy, Systematic biopsy, PRI-MUS, PI-RADS

## Abstract

Accurate assessment of tumor grade is critical for active surveillance (AS) in prostate cancer. We compared magnetic resonance imaging (MRI) and micro-ultrasound scoring (Prostate Imaging-Reporting and Data System [PI-RADS] v2.1 vs Prostate Risk Identification using Micro-ultrasound [PRI-MUS]) in 128 men on AS. The primary outcome was upgrading to Gleason grade group (GG) ≥2. There was no difference in GG ≥2 detection between the imaging techniques (PRI-MUS score ≥3: 33/34, 98%; PI-RADS score ≥3: 29/34, 85%; *p* = 0.22). The sensitivity, specificity, and positive and negative predictive values for GG ≥2 detection were 97%, 32%, 34%, and 97% with PRI-MUS ≥3, and 85%, 53%, 40%, and 91% with PI-RADS ≥3, respectively. Upgrading to GG ≥2 was more likely for PRI-MUS ≥3 than for PRI-MUS ≤2 scores (odds ratio 15.5, 95% confidence interval 2.0–118.5). A limitation is the lack of blinding to the MRI results. In conclusion, detection of upgrading to GG ≥2 during AS appears similar when using micro-ultrasound or MRI to inform prostate biopsy.

**Patient summary:**

We looked at a novel imaging technology, micro-ultrasound, in patients undergoing biopsy during active surveillance for prostate cancer. We found that micro-ultrasound can detect prostate cancer that may require treatment at a similar rate to that with magnetic resonance imaging (MRI) scans.

For men with low-risk prostate cancer (PCa), active surveillance (AS) has emerged as a standard-of-care option selected by >40% of patients [1], [2], [3]. AS limits risks to sexual, urinary, and bowel function in comparison to surgery and radiation [4]. The decision to opt for AS rather than undergo treatment is primarily based on tumor aggressiveness, so accurate assessment of this factor is key. In most AS programs, men enroll on the basis of a random, nontargeted biopsy revealing low-risk cancer (Gleason score 6, International Society of Urological Pathology grade group 1 [GG 1]) and undergo periodic biopsies to confirm that this low-risk categorization is maintained. However, many men (∼35% at 5 yr) will exhibit higher-risk PCa that requires active treatment [2], [3]. MRI-guided biopsy has been shown to improve detection of Gleason grade group ≥2 PCa as well as provide prognostication for future upgrading risk. Micro-ultrasound (microUS) is a recently developed advanced imaging tool that may assist in detection of PCa in real time during biopsy, but has yet to be studied in AS [5]. The objective of this study was to compare upgrading rates during AS using magnetic resonance imaging (MRI)-guided and microUS-guided biopsy.

The study cohort comprised 128 consecutive men previously diagnosed with any-volume GG 1 PCa who underwent combined MRI/microUS-guided transrectal fusion biopsy between September 2021 and February 2022 using an ExactVu MRI/microUS fusion device (Exact Imaging, Markham, Canada). Before biopsy, patients underwent multiparametric 3-T contrast-enhanced MRI and images were scored by two radiologists using the Prostate Imaging-Reporting and Data System (PI-RADS v2.1). For patients with a PI-RADS score ≥3, three MRI-targeted cores were obtained, followed by a 12-core systematic biopsy. At the time of biopsy, live microUS images for both targeted and systematic biopsies were given a Prostate Risk Identification using Micro-ultrasound score (PRI-MUS). Like the PI-RADS score, a PRI-MUS score of 1–2 was considered low risk and 3–5 suspicious for PCa.

The primary outcome was detection of clinically significant PCa (csPCa, defined as GG ≥2) stratified by PI-RADS and PRI-MUS scores. Statistical analyses were performed using χ^2^ tests, Fisher’s exact test, and McNemar’s test to compare csPCa detection rates. A two-sided *p* value of <0.05 was considered to be statistically significant.

PCa of any grade was diagnosed in 100/128 men (78%) and csPCa was identified in 34/128 (27%; Table 1 and Supplementary Table 1). There was no significant difference in csPCa detection between the imaging modalities: 33/34 men (98%) had a PRI-MUS score ≥3 and 29/34 (85%) had a PI-RADS score ≥3 (*p* = 0.22). Among the men diagnosed with any grade of PCa, 79/100 (79%) had a PRI-MUS score ≥3 and 64/100 (64%) had a PI-RADS score ≥3 (*p* < 0.001; Supplementary Table 2).

**Table 1:** Detection rate for clinically significant prostate cancer stratified by PRI-MUS and PI-RADS scores

	*n*	Grade group ≥2, *n* (%)	*p* value
Overall	128	34 (27)	
PRI-MUS score			
≤2	31	1 (3)	
3	36	10 (28)	**0.01**
4	37	9 (24)	**0.01**
5	24	14 (58)	**<0.001**
3–5	97	33 (34)	**0.001**
PI-RADS score			
≤2	55	5 (9)	
3	2	0 (0)	Not significant
4	39	17 (44)	**<0.001**
5	32	12 (38)	**0.001**
3–5	73	29 (40)	**<0.001**

PRI-MUS = Prostate Risk Identification using Micro-Ultrasound score; PI-RADS = Prostate Imaging-Reporting and Data System.

Men with a PRI-MUS score ≥3 were more likely to be diagnosed with csPCa than men with a PRI-MUS score ≤2 (*p* < 0.001; odds ratio [OR] 15.5, 95% confidence interval [CI] 2.0–118.5). Similarly, men with a PI-RADS score ≥3 were more likely to be upgraded to csPCa than men with a PI-RADS score ≤2 (*p* < 0.001; OR 6.6, 95% CI 2.4–18.5). Subjects were analyzed by biopsy indication (confirmatory vs surveillance biopsy) with similar results (Supplementary Tables 3 and 4).

The sensitivity, specificity, and positive and negative predictive values for csPCa detection were 97%, 32%, 34%, and 97% with PRI-MUS ≥3, and 85%, 53%, 40%, and 91% with PI-RADS ≥3, respectively (Table 2).

**Table 2:** Accuracy of micro-ultrasound and magnetic resonance imaging for detection of clinically significant prostate cancer

Imaging modality	Sensitivity (%)	Specificity (%)	PPV (%)	NPV (%)
Micro-ultrasound (PRI-MUS ≥3)	97	32	34	97
Magnetic resonance imaging (PI-RADS ≥3)	85	53	40	91

PRI-MUS = Prostate Risk Identification using Micro-Ultrasound; PI-RADS = Prostate Imaging-Reporting and Data System; PPV = positive predictive value; NPV = negative predictive value.

Only one man (3%) with a PRI-MUS score ≤2 and five men (9%) with a PI-RADS score ≤2 were upgraded to csPCa; no patient who was upgraded to csPCa had both a PRI-MUS score ≤2 and a PI-RADS score ≤2 (Supplementary Table 5).

MRI has improved detection of csPCa and decreased detection of clinically insignificant PCa [6]. However, MRI is not without limitations, including costs, accessibility, a learning curve, diagnostic delays due to the requirement for prebiopsy imaging, and contraindications (eg, renal impairment, ferromagnetic implants).

This study is among the first comparing microUS and MRI in AS. We showed that PRI-MUS scores ≥3 have a tenfold higher rate of csPCa detection in comparison to PRI-MUS scores ≤2. A PRI-MUS score ≥3 had 97% sensitivity for detection of csPCa. Smaller studies in AS have shown similar sensitivities for microUS, ranging from 84% to 93.3% [7], [8], [9], [10]. The sensitivity of MRI for csPCa detection in the present study was 85%, while other studies looking at microUS and MRI in the AS population reported sensitivity of 83–86.7% [8], [9].

In this study, patients underwent both MRI and microUS, with no difference in csPCa detection between the two imaging techniques. Importantly, all 34 of the patients in this study who were upgraded to csPCa had either a PRI-MUS score ≥3 or a PI-RADS score ≥3. Given the high sensitivity and high negative predictive values of an MRI- and microUS-guided approach, there is potential for eliminating the need for prostate biopsy in patients with a PRI-MUS score ≤2 and a PI-RADS score ≤2, but this will need to be confirmed in a further study. While all patients in our study diagnosed with csPCa had either a PRI-MUS score ≥3 or a PI-RADS score ≥3, ∼10% of these cases would have been missed if microUS were omitted.

Men undergoing confirmatory biopsy experience more upgrading events to csPCa (31%) than men undergoing continued surveillance biopsy (15%). Differences in upgrading rates between confirmatory and surveillance biopsies has previously been documented [3].

A limitation of our study is that the surgeon performing the microUS biopsy was not blinded to the MRI results. Another limitation is that MRI and microUS lesions were not targeted using the same method: microUS was used to identify lesions rather than target them. Furthermore, the surgeon has completed a formal course in microUS mastery, thereby limiting immediate generalization to all centers.

MicroUS-informed prostate biopsy could be a useful adjunct to help in detecting csPCa and could potentially prevent the need for MRI. A combination of MRI and microUS imaging results could potentially reduce the need for prostate biopsy in low-risk cases.

  ***Author contributions***: Adam Kinnaird had full access to all the data in the study and takes responsibility for the integrity of the data and the accuracy of the data analysis.

*Study concept and design*: Kinnaird, Wang, Fung, Albers.

*Acquisition of data*: Wang, Albers, Broomfield, Medina Martín.

*Analysis and interpretation of data*: Albers, Wang, Kinnaird.

*Drafting of the manuscript*: Albers, Kinnaird.

*Critical revision of the manuscript for important intellectual content*: Albers, Kinnaird.

*Statistical analysis*: Albers, Kinnaird.

*Obtaining funding*: Kinnaird.

*Administrative, technical, or material support*: Medina Martín.

*Supervision*: Kinnaird.

*Other*: None.

  ***Financial disclosures:*** Adam Kinnaird certifies that all conflicts of interest, including specific financial interests and relationships and affiliations relevant to the subject matter or materials discussed in the manuscript (eg, employment/affiliation, grants or funding, consultancies, honoraria, stock ownership or options, expert testimony, royalties, or patents filed, received, or pending), are the following: Adam Kinnaird has received grant funding from Exact Imaging for unrelated research studies. The remaining authors have nothing to disclose.

  ***Funding/Support and role of the sponsor*:** This study was supported by funding from the Alberta Cancer Foundation, Bird Dogs, and the University Hospital Foundation. Sponsors for this did not play a role in the collection or preparation of the manuscript.
