# Supplementary material for: Micro-ultrasound Versus Magnetic Resonance Imaging in Prostate Cancer Active Surveillance
Source: Eur Urol Open Sci. 2022 Oct 25;46:33–5. doi: 10.1016/j.euros.2022.09.019 (PMC9618766; doi:10.1016/j.euros.2022.09.019)
Supplement: Supplementary data 1 [file mmc1.docx]

**Supplementary material**

**Supplementary Table 1 – Baseline demographic, imaging, and biopsy characteristics for 128 patients with prostate cancer on active surveillance**

| Parameter | Result |
| --- | --- |
| Mean age, yr (standard deviation) | 65.8 (6.1) |
| Ethnicity, *n* (%)   Asian   Black   Caucasian   Hispanic   Indigenous   Unknown | 8 (6)  5 (4)  110 (86)  0 (0)  4 (3)  1 (1) |
| Family history of prostate cancer, *n* (%) | 47 (37) |
| Abnormal digital rectal examination, *n* (%) | 19 (15) |
| Median prostate-specific antigen, ng/ml (interquartile range) | 7.8 (5.3–10.7) |
| Median prostate volume, cm^3^ (interquartile range) | 57 (38–75) |
| Prostate-specific antigen density, *n* (%)   <0.15 ng/ml/cm^3^   ≥0.15 ng/ml/cm^3^ | 76 (59)  52 (41) |
| PRI-MUS score, *n* (%)   ≤2   3   4   5 | 31 (24)  36 (28)  37 (29)  24 (19) |
| PI-RADS score, *n* (%)   ≤2   3   4   5 | 55 (43)  2 (2)  39 (31)  32 (25) |
| Median number of targeted cores, *n* (interquartile range) | 3 (0–3) |
| Median number of systematic cores, *n* (interquartile range) | 12 (12–12) |
| Method for initial diagnosis, *n* (%)   Standard 12-core biopsy   Cognitive magnetic resonance imaging–guided biopsy   Transurethral resection of the prostate   Simple prostatectomy   Unknown | 104 (81)  11 (9)  8 (6)  2 (2)  3 (2) |
| Imaging before initial diagnosis, *n* (%)   Magnetic resonance imaging   Micro-ultrasound | 18 (14)  0 (0) |
| Median number of biopsies on active surveillance, *n* (interquartile range) | 1 (1–2) |
| Median follow-up, yr (interquartile range) | 2.4 (1.3–4.5) |

PRI-MUS = Prostate Risk Identification using Micro-Ultrasound score; PI-RADS = Prostate Imaging-Reporting and Data System.

**Supplementary Table 2 – Prostate cancer detection rate stratified by PRI-MUS and PI-RADS scores**

|  | *n* | Any cancer, *n* (%) | *p* value |
| --- | --- | --- | --- |
| Overall | 128 | 100 (78) |  |
| PRI-MUS score   ≤2   3   4   5   3–5 | 31  36  37  24  97 | 21 (68)  29 (81)  28 (76)  22 (92)  79 (81) | 0.23  0.47  **0.03**  0.11 |
| PI-RADS score   ≤2   3   4   5   3–5 | 55  2  39  32  73 | 36 (65)  2 (100)  33 (85)  29 (91)  64 (88) | 0.55  **0.04**  **0.01**  **0.003** |

PRI-MUS = Prostate Risk Identification using Micro-Ultrasound; PI-RADS = Prostate Imaging-Reporting and Data System.

**Supplementary Table 3 – Prostate cancer detection rates stratified by PRI-MUS and PI-RADS scores for men undergoing confirmatory biopsy**

|  | *n* | Any cancer, *n* (%) | *p* value | Grade group ≥2, *n* (%) | *p* value |
| --- | --- | --- | --- | --- | --- |
| Overall  PRI-MUS score   ≤2   3   4   5   3–5 | 89  21  22  28  18  68 | 73 (82)  16 (76)  18 (82)  22 (79)  17 (94)  57 (84) | 0.65  0.84  0.115  0.43 | 28 (31)  1 (5)  9 (41)  7 (25)  11 (61)  27 (40) | **0.005**  0.058  **<0.001**  **0.003** |
| Overall  PI-RADS score   ≤2   3   4   5   3–5 | 89  37  2  25  25  52 | 73 (82)  25 (68)  2 (100)  24 (96)  22 (88)  48 (92) | 1  **0.007**  0.06  **0.003** | 28 (31)  4 (11)  0 (0)  15 (60)  9 (36)  24 (46) | 1  **<0.001**  **0.02**  **<0.001** |

PRI-MUS = Prostate Risk Identification using Micro-Ultrasound; PI-RADS = Prostate Imaging-Reporting and Data System.

**Supplementary Table 4 – Prostate cancer detection rates stratified by PRI-MUS and PI-RADS scores for men undergoing ongoing surveillance biopsy**

|  | *n* | Any cancer, *n* (%) | *p* value | Grade group ≥2, *n* (%) | *p* value |
| --- | --- | --- | --- | --- | --- |
| Overall  PRI-MUS score   ≤2   3   4   5   3–5 | 39  10  14  9  6  29 | 27 (69)  5 (50)  11 (79)  6 (67)  5 (83)  22 (76) | 0.14  0.46  0.18  0.13 | 6 (15)  0 (0)  1 (7)  2 (22)  3 (50)  6 (21) | 1  0.21  **0.04**  0.31 |
| Overall  PI-RADS score   ≤2   3   4   5   3–5 | 39  18  0  14  7  21 | 27 (69)  11 (61)  0 (0)  9 (64)  7 (100)  16 (76) | 0.85  0.13  0.49 | 6 (15)  1 (6)  0 (0)  2 (14)  3 (43)  5 (24) | 0.4  **0.02**  0.12 |

PRI-MUS = Prostate Risk Identification using Micro-Ultrasound; PI-RADS = Prostate Imaging-Reporting and Data System.

**Supplementary Table 5 – Detection rate for clinically significant prostate cancer by PI-RADS and PRI-MUS score**

| Score combinations | Patients, *n* (%) | |
| --- | --- | --- |
|  | Grade group ≥2 | Grade group <2 |
| PI-RADS ≤2 and PRI-MUS ≤2 | 0 (0) | 23 (100) |
| PI-RADS 3–5 and PRI-MUS ≤2 | 1 (13) | 7 (87) |
| PI-RADS ≤2 and PRI-MUS 3–5 | 5 (16) | 27 (84) |
| PI-RADS 3–5 and PRI-MUS 3–5 | 28 (43) | 37 (57) |

PRI-MUS = Prostate Risk Identification using Micro-Ultrasound; PI-RADS = Prostate Imaging-Reporting and Data System.
